# Support vector machines predict postoperative memory outcomes in temporal lobe epilepsies

**DOI:** 10.1002/epi4.70119

**Published:** 2025-08-20

**Authors:** Silke Ethofer, Michael Erb, Monika Milian, Sabine Rona, Holger Lerche, Jürgen Honegger, Thomas Ethofer

**Affiliations:** ^1^ Department of Neurosurgery University Hospital Tübingen Tübingen Germany; ^2^ Department of Biomedical Magnetic Resonance University of Tübingen Tübingen Germany; ^3^ Department of Neurology and Epileptology University Hospital Tübingen Tübingen Germany; ^4^ Department of Psychiatry and Psychotherapy University Hospital Tübingen Tübingen Germany

**Keywords:** episodic memory, face‐name associations, fMRI, hippocampus, support vector machines, temporal lobe epilepsy

## Abstract

**Objective:**

We aimed to predict the side of epilepsy as well as the pre‐ and postoperative verbal and nonverbal memory performance in a cohort of left and right temporal lobe epilepsy (TLE) patients based on hippocampal activations during three different memory fMRI tasks (verbal, nonverbal and combined verbal and nonverbal) using support vector machines (SVM).

**Methods:**

Thirty‐five patients with unilateral TLE (20 left) were investigated before mesial temporal resection using a verbal, a nonverbal, and a face‐name (combined verbal and nonverbal) memory fMRI paradigm. SVM was used to test whether voxel‐by‐voxel activation patterns within the bilateral hippocampus during each of the three paradigms can be used to classify TLE patients according to their side of epilepsy, preoperative and postoperative verbal and nonverbal memory outcome.

**Results:**

Classification accuracy regarding the side of epilepsy was best for the face‐name paradigm closely followed by the verbal paradigm. Classification accuracy of the preoperative verbal memory performance was formally statistically significant for all three paradigms, but specificities were low. Regarding the preoperative nonverbal memory performance, activations during the nonverbal and the verbal paradigm provided high prediction accuracies. The results regarding the postoperative memory outcome revealed that activations during the verbal paradigm can be used to predict postoperative verbal memory outcome, whereas activations during the nonverbal paradigm can be used for the prediction of the nonverbal memory outcome. Preoperative activations during the face‐name paradigm were able to predict both the verbal and the nonverbal postoperative memory outcome.

**Significance:**

It is possible to classify TLE patients according to their side of epilepsy as well as their postoperative memory performance using SVM based on hippocampal activations during task‐based memory fMRI. The highest classification accuracies were obtained for the face‐name paradigm, suggesting this combined verbal and nonverbal paradigm to be most suitable to address these clinical questions. However, further validation in a larger cohort would be necessary.

**Plain Language Summary:**

This study aims to investigate whether it is possible to predict the side of epilepsy as well as the preoperative and postoperative verbal and nonverbal memory performance in left and right temporal lobe epilepsy patients using support vector machines (a machine learning technique) based on hippocampal activations during three different memory fMRI tasks. Results showed that classification of patients according to their side of epilepsy and their postoperative memory performance is possible, with the highest classification accuracies being obtained using a face‐name association task.

**Key points**

- SVM can classify TLE patients according to their side of epilepsy and postoperative memory performance.
- Classification accuracy regarding the side of epilepsy was best for the face‐name paradigm, closely followed by the verbal paradigm.
- Activations during the verbal paradigm can be used to predict postoperative verbal memory outcomes.
- Activations during the nonverbal paradigm can be used for the prediction of the nonverbal memory outcome.
- Activations during the face‐name paradigm could be used to predict both the verbal and the nonverbal postoperative memory outcomes.

## 1 INTRODUCTION

The most common type of epilepsy in adults is mesial temporal lobe epilepsy (mTLE) and selective resection represents an effective therapy for eliminating seizures or improving seizure control in 50–80% of patients.^1^, ^2^ However, as the hippocampus (HC) and associated mesial temporal structures are crucial for episodic memory processes, memory deficits are a frequent side effect of these resections.^3^ Especially, patients with left‐sided temporal surgery face a risk of 44% to their verbal memory as compared with 20% of patients undergoing right‐sided surgery.^3^ Therefore, the prediction of postoperative memory outcome in mTLE surgery is crucial during presurgical workup.

In addition to neuropsychological parameters, task‐based memory fMRI has been developed for the prediction of postoperative memory outcome in mTLE patients. A recent meta‐analysis revealed that memory fMRI based on laterality indices or symmetry of task activation is a modest predictor of outcomes in left TLE that should be considered in the context of a larger surgical workup.^4^ Most studies used material‐specific paradigms for verbal and nonverbal memory functions, for example, encoding of words to investigate left mesial temporal lobe (mTL) memory functions and abstract drawings for the assessment of right mTL memory functions.^4^ Using different paradigms to assess verbal and nonverbal memory functions is burdensome for the patients due to the necessity of longer scanning times. Paradigms that are able to activate both hippocampi within one run offer a certain advantage in this regard. Tackett et al. used a complex scene encoding task and were able to lateralize episodic memory functions in left and right TLE patients on an individual level.^5^ We developed a paradigm based on the encoding of face‐name associations to address both verbal and nonverbal memory functions equally^6^, ^7^ as face‐name associations elicit bilateral hippocampal activations in healthy subjects^6^, ^8^, ^9^ and can be employed to examine functional alterations of hippocampal activations in left and right TLE patients.^7^

Recently, the interest in machine learning (ML) techniques as diagnostic and prognostic tools has been growing. In epilepsy research, ML algorithms have been used for segmentation, lesion localization, lateralization, classification of epilepsy patients and healthy controls as well as for the prediction of postoperative seizure frequency (for reviews, see Ref. 10, 11). Applied ML algorithms relied on clinical data and neuropsychological scores,^12^, ^13^, ^14^ structural^15^, ^16^, ^17^, ^18^ and functional MRI data^19^ as well as combinations of different modalities.^15^, ^18^, ^20^, ^21^

However, studies using ML approaches such as support vector machines (SVM)^22^ for the prediction of postoperative memory outcome after mesial temporal resection are scarce. The present study relied on SVM using hippocampal activations from three memory paradigms (verbal, nonverbal and combined) as data to investigate its ability to classify (1) side of epilepsy, (2) preoperative verbal and nonverbal memory functions, and (3) postoperative verbal and nonverbal memory outcome in comparison to the individual lateralization index (LI). In addition, we aimed to evaluate whether the classification accuracy is higher for the combined paradigm or the material‐specific ones.

## 2 MATERIALS AND METHODS

### 2.1 Participants

We examined 35 German‐speaking patients with unilateral mTLE (20 left) with clear mesiotemporal spikes on EEG and typical temporal lobe seizure semiology. Twenty‐one patients had clear signs of unilateral hippocampal sclerosis on structural MRI as determined by experienced neuroradiologists, including unilateral hippocampal atrophy and increased T2 signal intensity; eight had temporo‐mesial tumors, one had a temporal focal cortical dysplasia, one a temporal cavernoma, one a reactive astrogliosis, and three patients showed no lesion on MRI. Nineteen of these patients underwent mTL resection (11 left). For further details regarding patients' characteristics, see Table 1. The study was approved by the Ethics committee of the University of Tübingen and was in accordance with the guidelines of the Declaration of Helsinki. All participants gave written informed consent.

**TABLE 1:** Demographic data of patients (*N* = 35).

Patients	Age/sex	Age at epilepsy onset (years)	Duration of epilepsy (years)	Seizure frequency (seizures/ month)	Epilepsy surgery performed yes/no	Etiology/type of lesion
**Left TLE group**						
1	30/F	15	15	8	No	Hippocampal sclerosis
2	36/M	14	22	6	No	Hippocampal sclerosis
3	49/F	1	48	20	Yes	Hippocampal sclerosis
4	34/F	28	6	4	No	No lesion
5	51/M	51	0.1	1	Yes	Temporo‐mesial tumor
6	34/F	30	4	300	Yes	Reactive astrogliosis
7	36/F	12	24	5	Yes	Hippocampal sclerosis
8	46/F	44	2	16	No	No lesion
9	46/M	17	29	12	Yes	Hippocampal sclerosis
10	23/F	23	0.3	0.25	No	Dysplasia
11	47/M	19	28	2	Yes	Hippocampal sclerosis
12	41/M	40	0.5	0.2	Yes	Temporo‐mesial tumor
13	25/F	15	10	5	Yes	Hippocampal sclerosis
14	57/F	22	35	20	Yes	Hippocampal sclerosis
15	49/F	33	16	6	No	Cavernoma
16	62/F	61	0	0.2	No	Temporo‐mesial tumor
17	28/F	19	9	4	Yes	Hippocampal sclerosis
18	66/M	66	0.25	0.3	Yes	Temporo‐mesial tumor
19	21/M	11	10	2	No	Hippocampal sclerosis
20	18/F	13	5	25	No	Hippocampal sclerosis
Mean (SEM)	40.0 (3.06)	26.7 (3.92)	13.2 (3.06)	21.8 (14.74)		
**Right TLE group**						
1	53/M	0	53	20	Yes	Hippocampal sclerosis
2	70/F	5	65	12	No	Hippocampal sclerosis
3	51/M	48	3	33	Yes	Hippocampal sclerosis
4	59/M	59	0.3	3	Yes	Temporo‐mesial tumor
5	64/M	4	60	1	No	Hippocampal sclerosis
6	57/F	45	12	13	No	Hippocampal sclerosis
7	51/M	10	41	30	No	Hippocampal sclerosis
8	21/M	5	16	28	Yes	Hippocampal sclerosis
9	17/M	16	1	20	Yes	Temporo‐mesial tumor
10	27/F	27	0.5	0.3	Yes	Temporo‐mesial tumor
11	44/M	44	0.1	0.1	No	Temporo‐mesial tumor
12	60/M	7	53	4	No	Hippocampal sclerosis
13	57/M	43	14	3	Yes	Hippocampal sclerosis
14	46/M	3	43	1	Yes	Hippocampal sclerosis
15	24/F	14	10	1	No	No lesion
Mean (SEM)	46.7 (4.30)	22.0 (5.23)	24.8 (6.35)	11.3 (3.09)		

Abbreviations: F, female; M, male; SEM, standard error of the mean; TLE, temporal lobe epilepsy.

### 2.2 Neuropsychological tests

Verbal learning and memory were evaluated using a wordlist learning and retention test which required memorizing a list of 15 words (Verbaler Lern‐ und Merkfähigkeitstest, VLMT^23^, ^24^). To assess nonverbal learning and memory, we used the revised version of the DCS (Diagnostikum für Cerebralschädigung^25^), during which subjects had to memorize nine geometrical figures. In both tests, we assessed the ‘immediate recall’ memory score, that is, the sum of correctly reproduced items during five learning trials. As memory performance levels decrease with age,^26^, ^27^ we report the percentile ranks of both memory tests for the analyses. The level of verbal crystallized intelligence was determined using the German multiple choice vocabulary test (MWT‐B, Mehrfachwahl‐Wortschatz‐Intelligenztest^28^, ^29^), which has been shown to correlate with the Full Scale IQ of the HAWIE‐R.^30^ In 14 of the 190 patients who underwent epilepsy surgery, memory scores were assessed at least 12 months postsurgery to guarantee postoperative memory consolidation (mean 17.8 months, SEM 1.85). The other five patients were lost to follow‐up.

### 2.3 Statistical analysis of behavioral data

Data were analyzed using IBM SPSS Statistics version 28 (http://www.spss.com). Descriptive statistics were used to analyze sociodemographic and neuropsychological characteristics using minimum, maximum, mean, and standard error of the mean (SEM). We calculated the percentages of correct answers for the fMRI task. Unfortunately, we did not obtain the responses in six patients (one left) due to technical difficulties. Differences between groups in behavioral performances were compared using two‐sample t‐tests. Differences in memory performance before and after epilepsy surgery were compared using paired t‐tests. The significance level was set at *p* < 0.05.

To enable classification by SVM, the patients were categorized according to their neuropsychological performance in the VLMT and the DCS in two groups: ‘below average’, that is, below the 16th percentile (≙ one standard deviation below the mean), and ‘average + above average.’

### 2.4 Acquisition of MRI data

MRI data were acquired using a Siemens Magnetom Sonata [Maestro Class] 1.5 T Scanner (Siemens AG) with an 8‐channel array head coil for reception and the body coil for transmission. A sagittal T1‐weighted 3D‐MPRAGE sequence was used to obtain a high‐resolution anatomical image of each subject's brain (TR/TI/TE = 1300/660/3.19 ms, flip angle 15°, field of view = 256 × 256 mm^2^, matrix 256 × 256, 176 slices, voxel size 1 ×1 × 1 mm^3^). A field map was recorded for distortion correction of the functional images caused by magnetic field inhomogeneity. For the fMRI tasks, gradient‐echo planar T2*‐weighted images covering the whole brain were acquired (TR = 4000 ms, TE = 64 ms, field of view = 192 × 192mm^2^, matrix 64 × 64, voxel size 3 × 3 × 3mm^3^, gap = 0.3 mm, 38 interleaved slices). All tasks were performed in block design with similar task designs to ensure inter‐task comparability. They consisted of 175 volumes each for the verbal and face‐name paradigm, and 110 volumes for the nonverbal paradigm. The first two images of each experimental run were discarded to reach equilibrium of magnetization.

### 2.5 Stimuli and fMRI tasks

#### 2.5.1 Verbal paradigm

To investigate verbal memory, subjects were to encode and recognize 24‐word pairs as described previously.^6^, ^31^, ^32^ The paradigm comprised six encoding blocks, each followed by a block of the control condition, in which subjects were presented with the names of two weekdays and had to indicate by button press whether they were identical or not. To ensure the participants' attention and compliance during the encoding condition, a recognition task (also alternating with blocks of the control task) was performed inside the scanner. For a more detailed description of the paradigm, see Ref. (6, 31, 32).

#### 2.5.2 Nonverbal paradigm

To investigate nonverbal memory functions, we used a spatial paradigm during which subjects were to encode and recognize object locations within a virtual 3D environment as described previously.^6^, ^33^, ^34^, ^35^ This paradigm comprised four encoding blocks, each followed by a control condition during which subjects were presented two differently sized versions of the objects and were asked to indicate by button press on which side the larger one was. The recognition task comprised four activation blocks alternating with four blocks of the control condition (detailed descriptions in Ref. 6, 33, 34, 35).

#### 2.5.3 Face‐name paradigm

To investigate verbal and nonverbal memory functions within one paradigm, we used a face‐name association task,^6^, ^7^ in which subjects were asked to memorize and recognize 24 face‐name pairs, alternating with a control condition in which two scrambled versions of the previously shown faces were presented, and subjects had to indicate by button press whether the two pictures were identical or not. This paradigm, too, comprised a two‐alternative forced‐choice recognition task alternating with the control condition. The detailed methods can be found in Ref. (6, 7).

The stimuli were presented using Presentation software (Neurbehavioral Systems Inc., http://www.neurobs.com) and were projected on a translucent screen positioned at the end of the scanner table via a video projector outside the magnet, and subjects saw them through a mirror attached to the head coil. They conveyed their responses via use of a two‐button box with their right thumb.

### 2.6 Statistical analysis of fMRI data

fMRI data were analyzed in MATLAB R2018b (http://www.mathworks.com) using Statistical Parametric Mapping (SPM12, Wellcome Trust Center for Imaging Neuroscience; http://fil.ion.ucl.ac.uk/spm). Each subject's imaging time series was corrected for differences in slice acquisition time, realigned, and unwarped based on the estimated field map data,^36^ co‐registered to the anatomical reference image, normalized to MNI space (Montreal Neurological Institute Atlas^37^), then smoothed with an isotropic Gaussian kernel (8 mm full‐with at half maximum) and filtered with a high‐pass filter with a cut‐off time of 128 s.

For first level analyses, experimental task and control blocks were defined by a box‐car function and convolved with the hemodynamic response function. Realignment parameters were added as regressors of no interest. Contrast images of individual main effects for the encoding versus control condition were calculated and second level analyses using one‐sample t‐tests were performed in order to examine task‐related group main effects. Results are reported at a height threshold of *p* < 0.001 (uncorrected). Correction for multiple comparisons (*p* < 0.05, corrected) across the whole brain was assessed at the cluster level using random field theory. The extent threshold for each contrast is reported in the results section.

To analyze hippocampal activations, a mask was created for the bilateral hippocampus using the automated anatomical labeling atlas (AAL^38^). This mask was then applied to each subject's first level results to extract beta estimates from both hippocampi, which were subsequently used as features in the SVM analyses. The correct fit of the mask was confirmed by visual inspection.

Additionally, we calculated lateralization indices (LI) for every contrast in each region of interest using the Bootstrap method of the SPM LI‐toolbox in order to obtain weighted mean values of lateralization.^39^ A lateralization index of < −0.65 or >0.65 was considered as strongly lateralized to the right or left hemisphere, respectively.

### 2.7 Support vector machines

SVM based on the LIBSVM toolbox^40^ were used to test whether voxel‐by‐voxel activation patterns within the hippocampi during each memory fMRI paradigm can be used to classify TLE patients according to their (1) side of epilepsy (left vs. right), (2) preoperative performance in the VLMT and the DCS (‘below average’ vs. ‘average + above average’) and (3) postoperative performance in the VLMT and the DCS (‘below average’ vs. ‘average + above average’). Due to the large number of features in our data sets, we used the linear kernel. The hyperparameter C was optimized by performing a grid search on the following values: *C* = 10^−3^, 10^−2^, 10^−1^, 10^0^, 10^1^, 10^2^, 10^3^, 10^4^, and the optimum C value for each analysis was determined. A leave‐one‐out cross‐validation procedure was employed, that is, the patient to be classified was not included in the training of the classifier. The performance of the classifier is reported using accuracy, sensitivity, and specificity. As we used a binary classifier, chance level was 50%. Accuracies were significantly above chance (*p* < 0.05), if they amounted to at least 65.71% for the preoperative analyses (*n* = 35) and 78.57% for the postoperative analyses (*n* = 15).

## 3 RESULTS

### 3.1 Neuropsychological performance

Comparison of demographic factors and neuropsychological test scores between left and right TLE patients revealed significant differences in the group of operated patients in their preoperative and postoperative VLMT and DCS scores, with left TLE patients performing worse than right TLE patients (preoperative VLMT: *t*(12)= −2.512, *p* = 0.027; preoperative DCS: *t*(6.8) = −2.547, *p* = 0.039; postoperative VLMT: *t*(5.7) = −4.698, *p* = 0.004; postoperative DCS: *t*(12)= −3.340, *p* = 0.006). Furthermore, we observed a tendency toward a worse performance of left TLE patients in the VLMT (*t*(33)= −1.616, *p* = 0.116), all other *p* > 0.05. For further details regarding the results of the neuropsychological tests, see Table 2. The left TLE group (*n* = 20) comprised nine patients with a VLMT performance below average, that is, the 16th percentile, and nine patients with a DCS performance below average. In the right TLE group (*n* = 15), there were two patients with a VLMT performance below average and five patients with a DCS performance below average. The operated group (*n* = 14) comprised seven patients with a VLMT performance below average (seven left) and six patients with a DCS performance below average (five left). In the operated left TLE group (*n* = 8), two patients declined in their verbal memory performance and three in their nonverbal memory performance. In the operated right TLE group (*n* = 6), only one patient declined in his nonverbal memory performance, while we observed no deteriorations in the patients' verbal memory performances.

**TABLE 2:** IQ and memory performance in LTLE and RTLE patients.

Group and variables	Minimum	Maximum	Mean (SEM)
LTLE (*n* = 20)			
IQ (MWT‐B)	79.0	124.0	99.2 (2.7)
VLMT PR	0.0	85.0	28.3 (7.0)
DCS PR	5.0	92.0	31.0 (6.9)
LTLE operated (*n* = 8)			
VLMT PR pre‐op	0.0	30.0	11.3 (4.4)
VLMT PR post‐op	0.0	20.0	5.0 (2.8)
DCS PR pre‐op	5.0	47.2	17.7 (5.7)
DCS PR post‐op	0.0	35.0	13.1 (4.5)
RTLE (*n* = 15)			
IQ (MWT‐B)	89.0	136.0	104.3 (4.2)
VLMT PR	0.0	90.0	44.3 (6.6)
DCS PR	0.0	81.5	44.1 (8.7)
RTLE operated (*n* = 6)			
VLMT PR pre‐op	0.0	90.0	40.8 (12.4)
VLMT PR post‐op	20.0	95.0	55.8 (10.4)
DCS PR pre‐op	5.0	81.5	54.6 (13.3)
DCS PR post‐op	8.6	81.5	47.7 (10.5)

Abbreviations: DCS, Diagnostikum für Cerebralschädigung; LTLE, left temporal lobe epilepsy; MWT‐B, Mehrfachwahl‐Wortschatz‐Intelligenztest (German multiple choice vocabulary test); PR, percentile ranks; RTLE, right temporal lobe epilepsy; VLMT, Verbaler Lern‐ und Merkfähigkeitstest (wordlist learning and memory test).

Comparison of preoperative and postoperative memory performances using paired samples t‐tests only revealed a statistically significant improvement in VLMT scores in the RTLE group (*t*(5)= −3.354, *p* = 0.020). All other comparisons showed no statistically significant differences, especially no significant postoperative memory loss (all *p* > 0.05).

Analysis of fMRI behavioral data revealed for the verbal paradigm 62.7 ± 5.24% correct responses for the left TLE group and 73.8 ± 7.67% for the right TLE group. For the nonverbal paradigm, the left TLE patients obtained 52.1 ± 4.77% correct responses and the right TLE patients 59.2 ± 7.15%. In the face‐name paradigm, left TLE patients recognized 69.5 ± 4.55% face‐name pairs correctly, and right TLE patients scored 75.1 ± 5.43% correct responses. There were no statistically significant differences between both groups (all *p* > 0.05).

### 3.2 fMRI whole brain results

**FIGURE 1:**
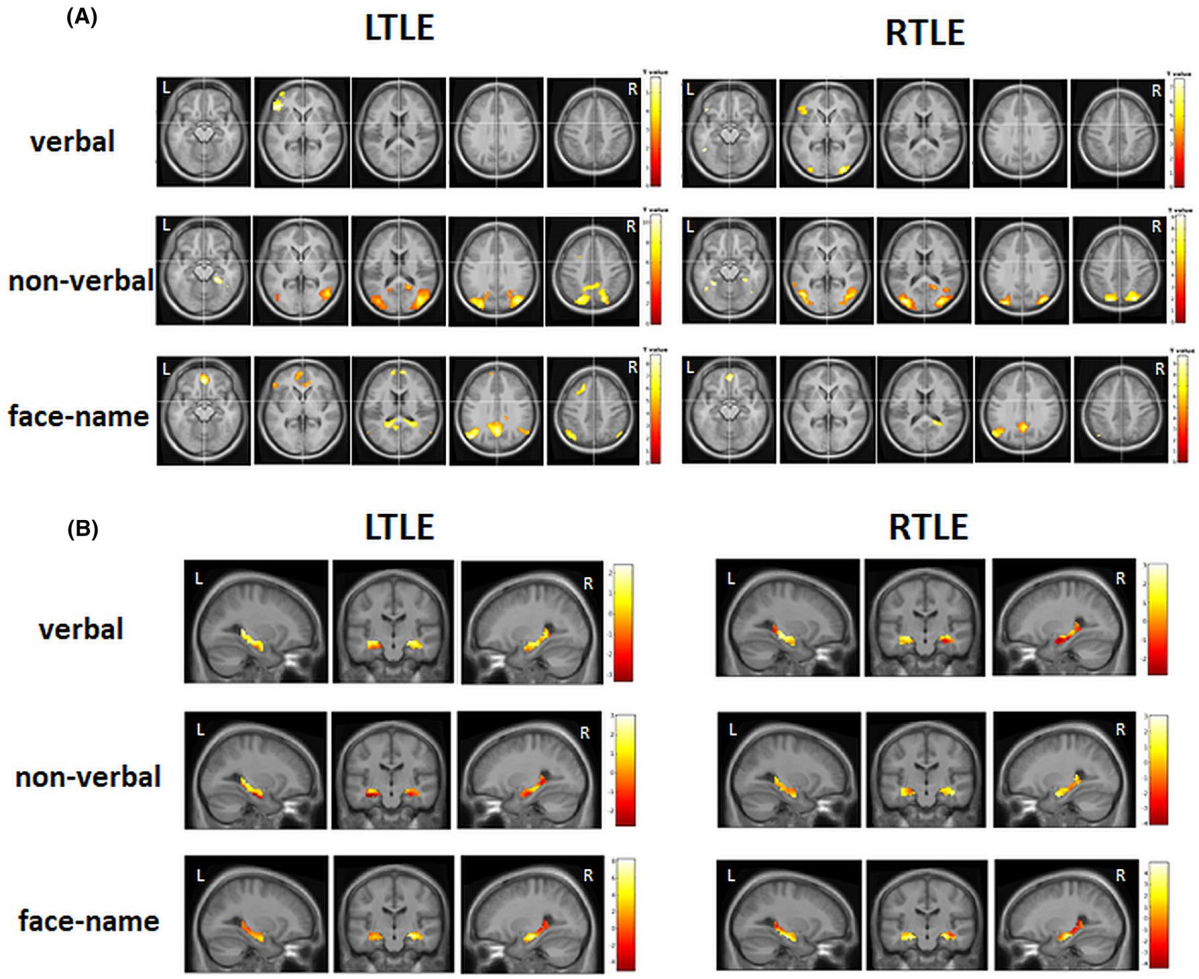
(A) fMRI activations in the left and right TLE group for the verbal, the nonverbal, and the face‐name paradigm (encoding > control condition), *p* < 0.05 FWE corrected at cluster level. Activations are projected on transversal slices (*z* = −15, *z* = 0, *z* = 15, *z* = 30, *z* = 45) of the mean normalized brain of the study participants, that is, the “mean” image created from all normalized structural images of our cohort. For further details regarding the activated brain regions/clusters, see Table 3. (B) Unthresholded fMRI activations within the hippocampal mask in the left and right TLE group for the verbal, the nonverbal, and the face‐name paradigm (encoding > control condition), which were used as features in the SVM analyses. Activations are projected on the mean normalized brain of the study participants.

The results of the second level analyses (encoding > control condition) are reported separately for the LTLE and RTLE groups and for the verbal, spatial, and face‐name paradigms in Table 3 and Figure 1.

**TABLE 3:** Activated brain regions/clusters.

Brain regions	MNI coordinates			Z score	Cluster size
**Verbal Paradigm**					
LTLE (*N* = 20)					
Frontal Inf Tri L, Frontal Inf Orb L, Frontal Mid L, Frontal Mid Orb L, Frontal Sup L	−54	39	9	4.40	219
RTLE (*N* = 15)					
Occipital Mid R, Occipital Inf R, Calcarine R	30	−93	0	4.69	107
Frontal Inf Tri L, Frontal Inf Orb L, Insula L	−42	33	−9	4.49	154
Occipital Mid L, Occipital Inf L	−30	−90	−3	4.17	47
Temporal Inf L, Fusiform L, Occipital Inf L	−45	−54	−12	3.98	52
*p < 0.05, FWE corrected, LTLE: *k* ≥ 219 voxels, RTLE: *k* ≥ 47 voxels					
**Spatial Paradigm**					
LTLE (*N* = 20)					
Occipital Mid R/L, Precuneus R/L, Temporal Mid R/L, Parietal Sup R/L, Occipital Sup R/L, Temporal Inf R, Angular R/L, Cuneus L/R, Parietal Inf L, Calcarine R/L, Cingulum Mid L/R, Occipital Inf R	39	−75	18	6.01	2771
Fusiform R, ParaHippocampal R, Lingual R	24	−39	−9	4.87	87
Frontal Mid L, Frontal Sup L	−27	9	51	4.12	63
RTLE (*N* = 15)					
Occipital Mid L, Parietal Sup L, Temporal Mid L, Occipital Sup L, Precuneus L, Temporal Inf L, Fusiform L, Parietal Inf L, Occipital Inf L, Lingual L, Angular L	−39	−81	12	5.96	1222
Occipital Mid R, Parietal Sup R, Temporal Mid R, Occipital Sup R, Precuneus R, Temporal Inf R, Fusiform R, Angular R, Lingual R, Cuneus R, Parietal Inf R, Calcarine R, ParaHippocampal R, Occipital Inf R	30	−84	18	5.32	1601
Frontal Mid L, Frontal Sup L	−36	9	57	4.72	48
*p < 0.05, FWE corrected, LTLE: *k* ≥ 63 voxels, RTLE: *k* ≥ 48 voxels					
**Face‐Name Paradigm**					
LTLE (*N* = 20)					
Frontal Med Orb L/R, Cingulum Ant L/R, Rectus L/R, Frontal Sup Medial L, Olfactory L	−3	42	−9	5.73	384
Angular L, Parietal Inf L, Occipital Mid L, Temporal Mid L	−48	−72	33	5.52	307
Precuneus L/R, Cingulum Mid L/R, Cingulum Post L/R, Cuneus L	−6	−57	27	4.96	597
Frontal Sup L, Frontal Mid L	−21	30	48	4.88	82
Frontal Sup L, Frontal Sup Medial L/R, Cingulum Ant R	−12	57	21	4.61	119
Frontal Inf Orb L, Frontal Inf Tri L, Frontal Mid Orb L	−45	33	−6	4.55	93
Angular R, Parietal Inf R, Temporal Mid R, Occipital Mid R, Temporal Sup R	51	−69	42	4.53	110
Precuneus R, Calcarine R, Cingulum Post R	12	−39	18	4.36	185
Cerebelum Crus 1 R, Cerebelum Crus 2 R	36	−75	−36	4.10	76
RTLE (*N* = 15)					
Angular L, Temporal Mid L, Occipital Mid L	−48	−72	27	5.28	185
Precuneus L/R, Cingulum Post L/R, Cingulum Mid L/R, Cuneus L	3	−57	30	4.74	173
Frontal Med Orb R/L, Cingulum Ant L/R, Rectus L/R	3	51	−12	4.71	158
Precuneus R, Calcarine R	18	−45	15	4.39	46
*p < 0.05, FWE corrected, LTLE: *k* ≥ 76 voxels, RTLE: *k* ≥ 46 voxels					

Abbreviations: Angular, angular gyrus; Calcarine, calcarine fissure and surrounding cortex; Cingulum Ant, anterior cingulate & paracingulate gyri; Cingulum Mid, middle cingulate & paracingulate gyri; Cingulum Post, posterior cingulate gyrus; Frontal Inf Orb, inferior frontal gyrus, orbital part; Frontal Inf Tri, inferior frontal gyrus, triangular part; Frontal Med Orb, superior frontal gyrus, medial orbital; Frontal Mid, middle frontal gyrus; Frontal Mid Orb, middle frontal gyrus, orbital part; Frontal Sup, superior frontal gyrus; Frontal Sup Medial, superior frontal gyrus, medial; Fusiform, fusiform gyrus; L, left; Lingual, lingual gyrus; Occipital Inf, inferior occipital gyrus; Occipital Mid, middle occipital gyurs; Occipital Sup, superior occipital gyrus; Olfactory, olfactory cortex; ParaHippocampal, parahippocampal gyrus; Parietal Inf, inferior parietal gyrus, excluding supramarginal and angular gyri; Parietal Sup, superior parietal gyrus; R, right; Rectus, gyrus rectus; Temporal Inf, inferior temporal gyrus; Temporal Mid, middle temporal gyrus; Temporal Sup, superior temporal gyrus.

Calculation of LI based on the results of the second level analyses for each paradigm in each patient group revealed in the LTLE group for the verbal paradigm a left‐sided lateralization (LI = 0.61), for the spatial paradigm a rather left‐sided lateralization (LI = 0.37), and for the face‐name paradigm a strongly right‐sided lateralization (LI = −0.71). LI within the RTLE group was strongly left‐sided for the verbal paradigm (LI = 0.88) and rather left‐sided for the spatial (LI = 0.41) and for the face‐name paradigm (LI = 0.59).

### 3.3 Classification using support vector machines and lateralization indices

**FIGURE 2:**
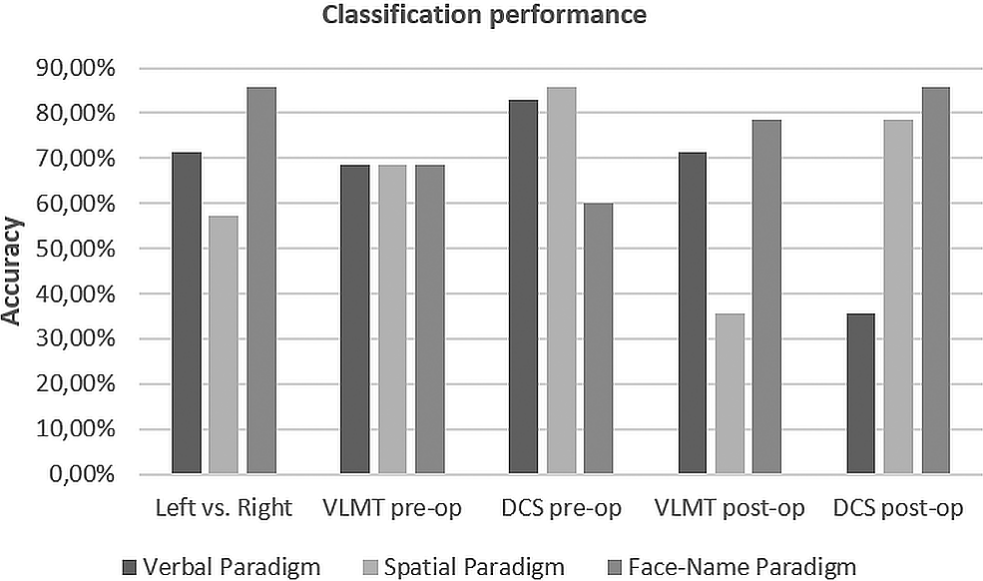
Overview of the classification performance of all three paradigms regarding side of epilepsy (Left vs. Right), preoperative verbal memory performance (VLMT pre‐op), preoperative nonverbal memory performance (DCS pre‐op), postoperative verbal memory performance (VLMT post‐op) and postoperative nonverbal memory performance (DCS post‐op). Significance level is 65.71% for “Left versus Right,” “VLMT pre‐op” and “DCS pre‐op” and 78.57% for “VLMT post‐op” and “DCS post‐op” (*p* < 0.05).

Classification accuracy regarding the side of epilepsy for the verbal paradigm was 71.43% (*p* = 0.008), for the spatial paradigm 57.14% (*p* = 0.250) and for the face‐name paradigm 85.71% (*p* < 0.001) (Table 4, Figure 2). Using the individual LIs, there are two possibilities of data interpretation: (1) Activations are stronger on the ipsilateral side, that is, the LI points to the ipsilateral side/the side of epilepsy. In this case, we obtained 34.3% (*p* = 0.980) correct classifications for the verbal paradigm, 42.9% (*p* = 0.845) for the spatial paradigm, and 37.1% (*p* = 0.955) for the face‐name paradigm. However, literature suggests reduced ipsilateral mesial temporal activations in TLE patients in memory fMRI.^7^ Therefore, it would be more plausible to hypothesize that (2) activations are stronger on the contralateral side, meaning that the LI would indicate the contralateral side. This would result in 65.7% (*p* = 0.045) correct classifications for the verbal paradigm, 57.1% (*p* = 0.250) for the spatial paradigm, and 62.9% (*p* = 0.088) for the face‐name paradigm (Table 4).

**TABLE 4:** Classification results for side of epilepsy and preoperative and postoperative verbal and nonverbal memory performances.

Pat.	Left vs. right			VLMT pre‐op			DCS pre‐op			VLMT post‐op			DCS post‐op		
	Verbal	Spatial	Face‐name	Verbal	Spatial	Face‐name	Verbal	Spatial	Face‐name	Verbal	Spatial	Face‐name	Verbal	Spatial	Face‐name
**Left TLE group (real data/predicted data with SVM/LI)**															
1	L/R/L	L/L/L	L/L/L	av/av/L	av/av/L	av/av/L	av/av/L	av/av/L	av/av/L	–	–	–	–	–	–
2	L/L/R	L/L/R	L/L/L	ba/av/R	ba/av/R	ba/ba/L	av/av/R	av/av/R	av/av/L	–	–	–	–	–	–
3	L/R/L	L/L/L	L/L/L	ba/av/L	ba/av/L	ba/av/L	ba/ba/L	ba/ba/L	ba/ba/L	–	–	–	–	–	–
4	L/R/L	L/L/L	L/L/L	av/av/L	av/av/L	av/ba/L	ba/ba/L	ba/av/L	ba/av/L	–	–	–	–	–	–
5	L/L/R	L/L/R	L/L/R	av/av/R	av/av/R	av/ba/R	av/av/R	av/av/R	av/av/R	–	–	–	–	–	–
6	L/L/R	L/L/R	L/R/R	av/av/R	av/av/R	av/av/R	ba/ba/R	ba/ba/R	ba/av/R	ba/ba/R	ba/av/R	ba/ba/R	av/av/R	av/av/R	av/av/R
7	L/L/L	L/L/R	L/L/R	av/av/L	av/av/R	av/av/R	ba/av/L	ba/ba/R	ba/ba/R	ba/ba/L	ba/av/R	ba/ba/R	av/ba/L	av/av/R	av/av/R
8	L/L/R	L/L/L	L/L/R	av/av/R	av/av/L	av/av/R	av/av/R	av/ba/L	av/av/R	–	–	–	–	–	–
9	L/L/L	L/L/L	L/L/R	ba/av/L	ba/av/L	ba/av/R	av/av/L	av/av/L	av/av/R	ba/ba/L	ba/av/L	ba/ba/R	ba/ba/L	ba/ba/L	ba/ba/R
10	L/L/R	L/L/R	L/L/L	av/av/R	av/av/R	av/av/L	av/av/R	av/av/R	av/ba/L	–	–	–	–	–	–
11	L/L/R	L/L/R	L/L/R	ba/av/R	ba/av/R	ba/av/R	ba/ba/R	ba/ba/R	ba/ba/R	ba/av/R	ba/ba/R	ba/ba/R	ba/av/R	ba/ba/R	ba/ba/R
12	L/L/L	L/L/R	L/R/R	ba/av/L	ba/av/R	ba/av/R	av/av/L	av/av/R	av/av/R	–	–	–	–	–	–
13	L/L/R	L/L/R	L/L/R	ba/av/R	ba/av/R	ba/av/R	ba/av/R	ba/ba/R	ba/av/R	ba/av/R	ba/ba/R	ba/ba/R	ba/av/R	ba/av/R	ba/ba/R
14	L/R/L	L/L/L	L/L/L	av/av/L	av/av/L	av/av/L	av/av/L	av/av/L	av/av/L	ba/ba/L	ba/av/L	ba/ba/L	av/ba/L	av/av/L	av/av/L
15	L/L/R	L/L/R	L/L/R	ba/av/R	ba/av/R	ba/ba/R	ba/ba/R	ba/av/R	ba/av/R	–	–	–	–	–	–
16	L/L/R	L/L/R	L/L/R	av/av/R	av/av/R	av/av/R	ba/ba/R	ba/av/R	ba/ba/R	–	–	–	–	–	–
17	L/L/L	L/L/L	L/L/R	ba/av/L	ba/av/L	ba/av/R	av/av/L	av/av/L	av/ba/R	ba/av/L	ba/av/L	ba/ba/R	ba/av/L	ba/av/L	ba/ba/R
18	L/R/L	L/L/R	L/L/R	av/av/L	av/av/R	av/ba/R	av/av/L	av/av/R	av/ba/R	av/av/L	av/ba/R	av/ba/R	ba/ba/L	ba/ba/R	ba/ba/R
19	L/L/R	L/L/L	L/L/L	ba/av/R	ba/av/L	ba/ba/L	ba/av/R	ba/av/L	ba/av/L	–	–	–	–	–	–
20	L/R/R	L/L/L	L/L/R	av/av/R	av/av/L	av/av/R	av/av/R	av/av/L	av/av/R	–	–	–	–	–	–
**Right TLE group (real data/predicted data with SVM/LI)**															
1	R/L/L	R/L/R	R/R/L	av/av/L	av/av/R	av/av/L	av/ba/L	av/av/R	av/av/L	av/av/L	av/ba/R	av/av/L	av/ba/L	av/av/R	av/av/L
2	R/L/R	R/L/R	R/L/L	av/av/R	av/av/R	av/av/L	ba/ba/R	ba/ba/R	ba/ba/L	–	–	–	–	–	–
3	R/R/R	R/L/L	R/R/L	ba/av/R	ba/av/L	ba/ba/L	av/av/R	av/ba/L	av/av/L	av/ba/R	av/av/L	av/av/L	av/ba/R	av/ba/L	av/av/L
4	R/R/R	R/L/R	R/R/R	av/av/R	av/av/R	av/av/R	av/av/R	av/av/R	av/av/R	–	–	–	–	–	–
5	R/R/L	R/L/L	R/R/L	ba/av/L	ba/av/L	ba/av/L	ba/ba/L	ba/ba/L	ba/av/L	–	–	–	–	–	–
6	R/R/L	R/L/L	R/L/R	av/av/L	av/av/L	av/av/R	av/av/L	av/av/L	av/av/R	–	–	–	–	–	–
7	R/R/L	R/L/R	R/R/L	av/av/L	av/av/R	av/av/L	ba/ba/L	ba/ba/R	ba/av/L	–	–	–	–	–	–
8	R/R/L	R/L/L	R/R/L	av/av/L	av/av/L	av/av/L	av/ba/L	av/av/L	av/av/L	av/av/L	av/av/L	av/ba/L	av/av/L	av/av/L	av/av/L
9	R/R/L	R/L/L	R/R/R	av/av/L	av/av/L	av/av/R	av/av/L	av/av/L	av/ba/R	av/av/L	av/av/L	av/av/R	av/av/L	av/av/L	av/ba/R
10	R/R/L	R/L/R	R/R/R	av/av/L	av/av/R	av/av/R	av/av/L	av/ba/R	av/av/R	–	–	–	–	–	–
11	R/R/L	R/L/L	R/R/R	av/av/L	av/av/L	av/av/R	av/av/L	av/av/L	av/av/R	–	–	–	–	–	–
12	R/L/L	R/L/L	R/R/R	av/av/L	av/av/L	av/av/R	ba/ba/L	ba/ba/L	ba/av/R	–	–	–	–	–	–
13	R/R/L	R/L/R	R/R/L	av/av/L	av/av/R	av/av/L	ba/av/L	ba/ba/R	ba/av/L	av/av/L	av/ba/R	av/av/L	ba/av/L	ba/ba/R	ba/av/L
14	R/R/L	R/L/L	R/R/L	av/av/L	av/av/L	av/av/L	av/av/L	av/av/L	av/ba/L	av/av/L	av/ba/L	av/ba/L	av/ba/L	av/av/L	av/av/L
15	R/L/L	R/L/L	R/L/L	av/av/L	av/av/L	av/ba/L	av/av/L	av/av/L	av/av/L	–	–	–	–	–	–
**Acc. SVM/LI (%)**	71.4*/34.3	57.1/42.9	85.7**/37.1	68.6*/37.1	68.6*/51.4	68.6*/51.4	82.9**/51.4	80.0**/48.6	60.0/54.3	71.4(*)/35.7	35.7/42.9	78.6*/57.1	35.7/42.9	78.6*/35.7	85.7*/57.1
**Sens. SVM/LI (%)**	70.0/45.0	100/45.0	90.0/35.0	100/50.0	100/50.0	83.3/50.0	90.5/60.0	85.7/60.0	76.2 50.0	85.7/50.0	42.9/50.0	57.1/75.0	37.5/50.0	87.5/50.0	87.5/75.0
**Spec. SVM/LI (%)**	73.3/20.0	0/40.0	80.0/40.0	0/20.0	0/53.3	36.4/53.3	71.4/40.0	71.4/33.3	35.7/60.0	57.1/16.7	28.6/33.3	100/33.3	33.3/33.3	66.7/16.7	83.3/33.3

*Note*: **p < 0.001, *p < 0.05, (*)*p* < 0.1.

Abbreviations: Acc., Accuracy; av., average memory performance; ba, below average memory performance; DCS, Diagnostikum für Cerebralschädigung; L, left TLE; L, left; LI, lateralization index; R, right TLE; R, right; Sens., sensitivity; Spec., specificity; TLE, temporal lobe epilepsy; VLMT, Verbaler Lern‐ und Merkfähigkeitstest (wordlist learning and memory test).

Regarding the preoperative verbal memory performance (VLMT), classification accuracy was 68.57% (*p* = 0.020) for the verbal paradigm, 68.57% (*p* = 0.020) for the spatial paradigm, and 68.57% (*p* = 0.020) for the face‐name paradigm (Table 4, Figure 2). Based on the individual LI and hypothesizing that ipsilateral lateralization indicates average and above average performances, we observed 37.1% (*p* = 0.955) correct classifications for the verbal paradigm, 51.4% (*p* = 0.500) for the nonverbal paradigm, and 51.4% (*p* = 0.500) for the face‐name paradigm. Hypothesizing that contralateral lateralization indicates average and above average performance, we obtained 62.9% (*p* = 0.088) correct classifications for the verbal paradigm, 48.6% (*p* = 0.632) for the nonverbal paradigm, and 48.6% (*p* = 0.632) for the face‐name paradigm (Table 4).

Classification accuracy for the preoperative nonverbal memory performance (DCS) was 82.9% (*p* < 0.001) for the verbal paradigm, 80.0% (*p* < 0.001) for the spatial paradigm, and 60% (*p* = 0.155) for the face‐name paradigm (Table 4, Figure 2). Using the individual LIs, we obtained 51.4% (*p* = 0.500) correct classifications for the verbal paradigm, 48.6% (*p* = 0.632) for the nonverbal paradigm, and 54.3% (*p* = 0 368) for the face‐name paradigm, hypothesizing that ipsilateral lateralization indicates average and above average performances, and 48.6% (*p* = 0.632), 51.4% (*p* = 0.500) and 45.7% (*p* = 0 750) for the verbal, nonverbal, and face‐name paradigms, respectively, hypothesizing that contralateral lateralization indicates average and above average performance (Table 4).

Regarding the prediction of postoperative memory outcome, classification accuracy for the verbal memory scores (VLMT) was 71.4% (*p* = 0.090) for the verbal paradigm, 35.71% (*p* = 0.910) for the spatial paradigm, and 78.6% (*p* = 0.029) for the face‐name paradigm (Table 4, Figure 2). Using the individual LIs, we observed 35.7% (*p* = 0.910), 42.9% (*p* = 0.788) and 57.1% (*p* = 0,395) correct classifications for the verbal, nonverbal, and face‐name paradigms if ipsilateral activations are hypothesized to indicate average and above average performance, and 64.3% (*p* = 0.212), 57.1% (*p* = 0.395) and 42.9% (*p* = 0.788) for the verbal, nonverbal, and face‐name paradigms, respectively, if contralateral activations are hypothesized to indicate average and above average performance (Table 4).

Classification of the postoperative nonverbal memory outcome (DCS) revealed an accuracy of 35.71% (*p* = 0.910) for the verbal paradigm, 78.6% (*p* = 0.029) for the spatial paradigm, and 85.7% (*p* = 0.006) for the face‐name paradigm (Table 4, Figure 2), while 42.9% (*p* = 0.788), 35.7% (*p* = 0.910), and 57.1% (*p* = 0.395) correct classifications were achieved using the individual LIs of the verbal, nonverbal, and face‐name paradigm if ipsilateral activations are hypothesized to indicate average and above average performance, and 57.1% (*p* = 0.395), 64.3% (*p* = 0.212) and 42.9% (*p* = 0.788) if contralateral activations are hypothesized to indicate average and above average performance (Table 4).

## 4 DISCUSSION

We investigated whether SVM applied to hippocampal activations during three different memory paradigms (material‐specific vs. combined) can be used to classify patients according to their side of epilepsy as well as their pre‐ and postoperative verbal and nonverbal memory performance. Classification accuracy regarding the side of epilepsy was best for the face‐name paradigm closely followed by the verbal paradigm. Classification accuracy of the preoperative verbal memory performance was formally statistically significant for all three paradigms but specificities were low. Regarding the preoperative nonverbal memory performance, not only activations during the nonverbal, but also the verbal paradigm were useful features for the classifier with high prediction accuracies. The results regarding the postoperative memory outcome revealed that activations during the verbal paradigm can be used to predict postoperative verbal memory outcome, whereas activations during the nonverbal paradigm can be used for the prediction of the nonverbal memory outcome. Activation patterns obtained during the face‐name paradigm could be used to predict both the verbal and the nonverbal postoperative memory outcome. In contrast, classifications based on the individual LIs were only significantly above chance for the verbal paradigm regarding the side of epilepsy; all other classifications did not reach statistical significance, that is, the performance of the LI lies clearly below that of the SVM analyses. This underlines the superiority of SVM regarding its ability to correctly identify the side of epilepsy as well as the pre‐ and postoperative verbal and nonverbal memory performance in a cohort of right‐ and left‐sided TLE patients.

### 4.1 Pre‐ and postoperative memory performances

Interestingly, comparison of left and right TLE patients regarding their verbal and nonverbal memory performance revealed no statistically significant differences between groups, although there seems to be a tendency toward a lower performance in the left TLE group regarding the verbal memory score. This would be in accordance with existing literature, as several studies have demonstrated that patients with left‐sided TLE perform significantly worse in verbal memory tests compared with patients with right‐sided TLE,^41^, ^42^, ^43^ while there are no significant differences regarding nonverbal memory performance,^41^ as is the case in our cohort. The reason for the fact that left TLE patients are also considerably impaired on the DCS most probably lies in the verbalizability of visual material. It has been shown that verbalization can both improve and hinder visual memory performance. RTLE patients are thought to rely on “left hemispheric,” that is, verbal, capacities for the encoding of visual material to improve performance. LTLE patients, on the other hand, might perform worse in nonverbal memory tests due to poorly encoded verbalizations as a result of their verbal memory deficits.^44^, ^45^ The DCS is a nonverbal memory test in which items can be named, but the information content exceeds verbal memory capacity to reduce this major confounding factor.^44^ But apparently in patients with memory deficits, either verbal or nonverbal, this factor still comes into play.^35^

Regarding the subgroup of operated patients, left TLE patients performed worse in both memory tests than right TLE patients. As it is generally acknowledged that resection within the mTL bears the risk of significant decline in episodic memory functions, especially on the left side^3^, ^46^, ^47^ and that patients with good memory abilities prior to surgery are more likely to decline,^46^, ^48^ we are reluctant to perform mTL resections in patients with good memory functions, especially in the left hemisphere. Therefore, this discrepancy in the operated group with regard to the whole cohort is due to the fact that mainly left TLE patients with a low memory performance prior to surgery underwent mTL resections.

### 4.2 Classification regarding the side of epilepsy

Employing SVM, we were able to classify left versus right TLE patients with an accuracy of 85.71% using bilateral hippocampal activations during a face‐name association task. Classification accuracy using activations during the verbal paradigm was slightly lower, but still clearly above chance at 71.43%. Using the individual LI for classification, the results were only significantly above chance for the verbal paradigm at 65.7%, not for the spatial and the face‐name paradigm. This indicates that hippocampal activation patterns in both the face‐name and – to a lesser extent – the verbal paradigm are able to differentiate between left and right TLE, especially when using SVM for classification. In the existing literature, features derived from different modalities have been used to classify TLE patients according to their side of epilepsy. Roger et al.^14^ used different neuropsychological scores and obtained predictive classification performances >75%, whereas lateralization accuracy in the study of Frank and colleagues^13^ reached only 61.7%. However, the latter included not only TLE but also extra temporal epilepsies in their study. Several groups used features derived from structural MRI images to lateralize the epileptogenic focus in TLE patients^49^, ^50^, ^51^, ^52^, ^53^, ^54^, ^55^, ^56^, ^57^, ^58^ with accuracies ranging from 56%^58^ up to 100% in patients with imaging evidence for hippocampal sclerosis^55^ (for reviews, see^10^, ^59^). One study used a machine learning‐based method with features extracted from resting‐state functional connectivity data in a cohort of 12 TLE patients and achieved a prediction accuracy of 83%.^19^ The results obtained with our task‐based memory paradigms, especially the face‐name paradigm, are in line with all these studies. Interestingly, calculations of LIs based on the results of the fMRI group analyses within the hippocampal ROI showed for the face‐name paradigm a clear lateralization to the contralateral side in both patient groups, thereby indicating the side of epilepsy and supporting the finding that this paradigm can be used to investigate TLE patients irrespective of the side of the seizure focus. However, on a single‐subject level, classification based on the individual LIs using the face‐name paradigm was only successful in 62.9% of cases, which is just below the statistical significance threshold (*p* = 0.088).

Furthermore, our paradigms are not only able to classify TLE patients according to their side of epilepsy, but they also shed light on hippocampal activations during verbal and nonverbal episodic memory processing, the networks involved in episodic memory functions, and alterations in left and right TLE patients.^6^, ^7^, ^32^, ^35^, ^60^

### 4.3 Classification of preoperative memory performance

Making use of the ability of our paradigms to depict memory functions, we investigated to what extent hippocampal activations can be used to identify TLE patients with bad memory performance, that is, to classify them according to their memory functions in ‘below average’ and ‘average + above average.’ Regarding the verbal memory performance, classification accuracies for all three paradigms were with 68.57% each formally statistically significant with high sensitivities of 83.3%–100%. Specificities, however, were low (0–36.4%). Classification accuracies for the nonverbal memory performances were statistically significant for the nonverbal paradigm with 80% and a sensitivity of 85.7% and a specificity of 71.4%. Interestingly, hippocampal activations during the verbal paradigm could also be used to classify TLE patients with respect to their nonverbal memory functions with an even higher accuracy of 82.9% as well as a sensitivity of 90.5% and a specificity of 71.4%. Classifications based on the LI did not reach statistical significance. So far, the majority of studies on task‐based memory fMRI in TLE have focused on correlations between BOLD activations and memory functions assessed by neuropsychological testing.^6^, ^7^, ^32^, ^35^, ^61^, ^62^, ^63^, ^64^, ^65^, ^66^, ^67^ The current study represents the use of ML and a dichotomous classification to identify patients with below average performance using a clear cut‐off value (16th percentile). While classification regarding the patients' nonverbal memory performances seems very well possible with our verbal and nonverbal paradigms, classification of patients according to their verbal memory functions is still difficult as our paradigms were not able to identify patients with below average verbal memory based on hippocampal activations. A possible reason for this might be the rather small cohort with the resulting small training data set.

### 4.4 Prediction of postoperative memory outcome

The main interest in task‐based memory fMRI, however, lies in the prediction of the postoperative memory outcome after mesial temporal resections. A recent meta‐analysis has investigated its predictive validity, indicating that memory fMRI based on laterality indices or symmetry of task activation is a modest predictor of memory outcomes in left TLE and should be considered in the context of a larger surgical workup.^4^ To the best of our knowledge, our study is the first using ML algorithms on task‐based memory fMRI to predict the postoperative memory outcome. Using a face‐name paradigm, which is designed to combine verbal and nonverbal memory functions, we were able to classify patients according to both their verbal memory functions with an accuracy of 78.6% and their nonverbal memory functions with an accuracy of 85.7%. Using hippocampal activations during the verbal paradigm, SVM were able to classify patients according to their postoperative verbal memory score with an accuracy of 71.4%, whereas activations from the nonverbal paradigm could be used to classify patients according to their nonverbal postoperative memory scores in 78.6% of cases. Again, classifications based on the LI did not reach statistical significance. Ljunggren et al.^68^ developed a model to identify patients with postoperative decline in verbal memory based on the side of surgery, inclusion or not of the hippocampus in the resection, preoperative verbal memory function, and presence or absence of focal to bilateral tonic–clonic seizures, which was able to correctly identify 82% of patients with postoperative memory decline. Using a predictive ML approach based on preoperative neuropsychological indices, Roger et al.^69^ were able to classify TLE patients according to their long‐term postoperative naming outcome in about 2/3 of cases. Busch and collaborators^70^ reported reliable prediction of postoperative verbal memory decline including the side of surgery, baseline memory score, hippocampal resection, and educational level. However, all these models are based on clinical data. We used task‐based memory fMRI, which offers additional possibilities by providing information about the functionality of the hippocampus to be resected and by delineating the network involved in episodic memory functions and its changes in TLE patients.^7^, ^35^

Interestingly, SVM based on hippocampal activations during the face‐name paradigm was able to classify patients both according to their verbal and their nonverbal episodic memory functions. In a previous study, we were able to demonstrate that this face‐name association task can be employed to examine functional alterations during the encoding of both verbal and nonverbal stimuli in one fMRI paradigm demonstrating diminished activations within the hippocampus depending on the side of epilepsy in a cohort of TLE patients with hippocampal sclerosis.^7^ This paradigm was designed to address both verbal and nonverbal memory functions as face‐name associations have been shown to rely on both mTLs and elicit bilateral hippocampal activations in healthy subjects.^7^, ^8^, ^9^ Similar observations have been made by Tackett and colleagues who used a complex scene encoding task to lateralize episodic memory functions in left and right TLE patients on an individual level.^5^ These bilateral paradigms enable the investigator to address both verbal and nonverbal memory functions using one single fMRI paradigm, which has the advantage of being faster and easier to apply in everyday clinical routine than two material‐specific paradigms, and thus represents an additional gain in the presurgical evaluation of TLE patients.

### 4.5 Limitations

The main limitation of our study is the rather small sample size, especially regarding the subgroup of operated patients. With regard to the application of SVM, the main problem associated with smaller sample sizes is the problem of overfitting. We tried to prevent this using a leave‐one‐out cross‐validation. However, a larger set of training data and an independent data set for validation would be desirable.

Furthermore, the small cohort made separate SVM analyses of certain subgroups like patients experiencing changes in their postoperative memory performance vs. those who do not, nonlesional patients versus lesional patients, and left versus right TLE patients, impossible due to the resulting insufficient training data set. Regarding the latter, it is, however, important to keep in mind that the side of epilepsy cannot always be clearly identified in TLE patients, especially in patients with nonlesional MRIs. According to our results, the prediction of memory functions is possible even in ignorance of the side of epilepsy as we pooled left and right TLE for our analyses. Whether this holds true for bilateral TLE patients needs to be evaluated in a separate study.

### 4.6 Clinical relevance

We were able to classify TLE patients according to their side of epilepsy as well as their postoperative verbal and nonverbal memory performance using SVM based on hippocampal activations during memory fMRI. The face‐name paradigm proved to be superior to the two material‐specific paradigms as it was the only paradigm to answer all three questions about laterality and the postoperative verbal and nonverbal memory outcome with high accuracy. The use of only one paradigm in clinical routine saves time and is more comfortable for the patients. Therefore, the face‐name paradigm is better suited to address the clinical questions examined here than two separate material‐specific paradigms, highlighting its role in the presurgical workup of epilepsy patients.

## FUNDING INFORMATION

This work was supported by the fortüne‐Program (2055‐0‐1 and 2343‐0‐0) of the University of Tübingen.

## CONFLICT OF INTEREST STATEMENT

None of the authors has any conflicts of interest to disclose.

## ETHICS STATEMENT

We confirm that we have read the Journal's position on issues involved in ethical publication and affirm that this report is consistent with those guidelines.

## Data Availability

The data that support the findings of this study are available from the corresponding author upon reasonable request.
